# Arthropod diversity in two Historic Gardens in the Azores, Portugal

**DOI:** 10.3897/BDJ.8.e54749

**Published:** 2020-08-06

**Authors:** Alba Arteaga, Jagoba Malumbres-Olarte, Rosalina Gabriel, Alejandra Ros-Prieto, Pedro Casimiro, Ana Fuentes Sanchez, Isabel S. Albergaria, Paulo A.V. Borges

**Affiliations:** 1 CE3C – Centre for Ecology, Evolution and Environmental Changes / Azorean Biodiversity Group and Universidade dos Açores, Angra do Heroísmo, Azores, Portugal CE3C – Centre for Ecology, Evolution and Environmental Changes / Azorean Biodiversity Group and Universidade dos Açores Angra do Heroísmo, Azores Portugal; 2 LIBRe – Laboratory for Integrative Biodiversity Research, Finnish Museum of Natural History, University of Helsinki, Helsinki, Finland LIBRe – Laboratory for Integrative Biodiversity Research, Finnish Museum of Natural History, University of Helsinki Helsinki Finland; 3 Jardim Botânico do Faial, Sociedade de Gestão Ambiental e Conservação da Natureza, Azorina S.A., Horta, Azores, Portugal Jardim Botânico do Faial, Sociedade de Gestão Ambiental e Conservação da Natureza, Azorina S.A. Horta, Azores Portugal; 4 Universidade dos Açores, CHAM e FCSH Rua Mãe de Deus, 9500-321, Ponta Delgada, Azores, Portugal Universidade dos Açores, CHAM e FCSH Rua Mãe de Deus, 9500-321 Ponta Delgada, Azores Portugal

**Keywords:** Arthropods, Araneae, Coleoptera, Hemiptera, diversity metrics, Hill series, beta diversity partitioning, urban gardens, introduced species, endemic species

## Abstract

The aim of our study was to characterise and compare the richness and composition of endemic, native (non-endemic) and introduced arthropod assemblages of two Azorean Historic Gardens with contrasting plant species composition. We hypothesised that Faial Botanic Garden would hold higher arthropod diversity and abundance of native and endemic arthropod species due to its larger native plant community. Species were collected using several arthropod standardised techniques between April 2017 and June 2018. We used the alpha diversity metrics (Hill series) and the partitioning of total beta diversity (β_total_) into its replacement (β_repl_) and richness (β_rich_) components, to analyse the adult and total arthropod community. The orders Araneae, Coleoptera and Hemiptera were also studied separately. Our results show that the number of exotic arthropod species exceeds the number of native and/or the endemic species in both gardens, but the arthropod community of Faial Botanic Garden exhibited a higher density of endemic and native species. Despite some minor exceptions, the geographic origins of plant communities largely influenced the arthropod species sampled in each garden. This study improves our knowledge about urban arthropod diversity in the Azores and shows how well-designed urban garden management and planning contribute to the conservation of native and endemic Azorean species.

## Introduction

Urban population has rapidly increased from 751 million in 1950 to 4.2 billion in 2018 (Anonymous 2018). Moreover, the United Nations estimates that, by 2050, almost two thirds (68%) of the world population will live in urban areas (Anonymous 2018). The development and expansion of cities into rural and natural areas has a detrimental effect on diversity (Sattler et al. 2010). Indeed, one of the main drivers of biodiversity erosion is land-use changes promoted by urbanisation (Borges et al. 2019a). These land-use changes are degrading and fragmenting native habitats (including underground ecosystems, as lava tubes on volcanic areas), which negatively affect species survival. Urbanisation facilitate the establishment of introduced species and several patterns may occur (McKinney 2008): i) medium levels of urbanisation seem to increase species richness because of greater heterogeneity and co-occurrence of generalist species; ii) high levels of urbanisation may decrease biological diversity drastically due to local extinction of disturbed sensitive species.

It is widely accepted that the design and planning of urban gardens may play an important role in species conservation due to the influence of plant species on the structure and composition of the local arthropod community (Smith et al. 2005). Many gardens house ornamental exotic plant species that could act as a pathway for arthropod (Dawson et al. 2008, Ward and Amatangelo 2018) and plant (Gabriel 2019) invasions which endanger native flora and wildlife (Reichard and White 2001). It is also shown that introduced generalist species may be more likely to colonise urban places compared to native specialists (Matteson et al. 2008, Kowarik 2011). On the other hand, public gardens that contain native plant species become part of native flora networks, facilitating genetic exchange amongst isolated populations (Roberts et al. 2007) and connecting native arthropod populations.

The native flora and fauna of the archipelago of the Azores (Portugal) have undergone profound changes and alterations since Portuguese explorers discovered and occupied these islands in the early fifteenth century (Santos et al. 2003, Norder et al. 2020). Despite the fact that human population in Azores is concentrated in coastal areas, the impact of deforestation on the native laurel forest has been extensive and, currently, it only covers 2-5% of its original area (Gaspar et al. 2008, Norder et al. 2020). In the second half of the 20th century, the loss and degradation of the native habitats is mainly driven by the adopted economic model, which relies heavily on dairy farming and fisheries (Norder et al. 2020). Increasing agricultural and livestock practices have led to a substantial alteration of the native flora via habitat fragmentation, changes in land use and the introduction of exotic species, which are a serious threat to the Azorean wildlife (Martins 1993, Silva et al. 2008, Borges et al. 2017a). As major land-use changes and urbanisation continue in the Azores, the creation or maintenance of “green spaces” within cities, such as private domestic gardens and public gardens, could help minimise the impact of urbanisation, at least for lowland native arthropod communities. Indeed, the nine Azorean islands sustain more than 2300 species and subspecies of terrestrial arthropods, being 42% introduced, 32% native non-endemic and only 12% endemic to the archipelago (Rego et al. 2015). Moreover, a large fraction of the 123 endemic arthropods recently assessed by IUCN were classified as Critically Endangered (32%), Endangered (27%) or Vulnerable (7%), with only 27% being classified in the lower conservation categories (NT, LC or DD) (Borges et al. 2017c, Borges et al. 2018a, Borges et al. 2019b, IUCN 2020).

In this study, we focused on two public gardens at low elevation, with contrasting features: one public garden designed to include mostly endemic and native flora from the Azores (the Faial Botanic Garden); and a public garden designed to host mainly exotic plants from all over the world (Duque da Terceira Garden). The aim of our study was to characterise and compare the richness and composition of endemic, native non-endemic and introduced arthropod assemblages of each garden by testing the following two hypotheses: (i) due to the origin of the plants, the species richness and abundance of endemic and native non-endemic arthropods should be higher at the Faial Botanic Garden and that (ii) the richness and abundance of introduced species should be higher at Duque da Terceira Garden. This information could be crucial for the design, planning and management of future models of urban gardens in the Azores for a better contribution to their biodiversity conservation.

## Material and methods

### Study Sites

**Figure 1.**
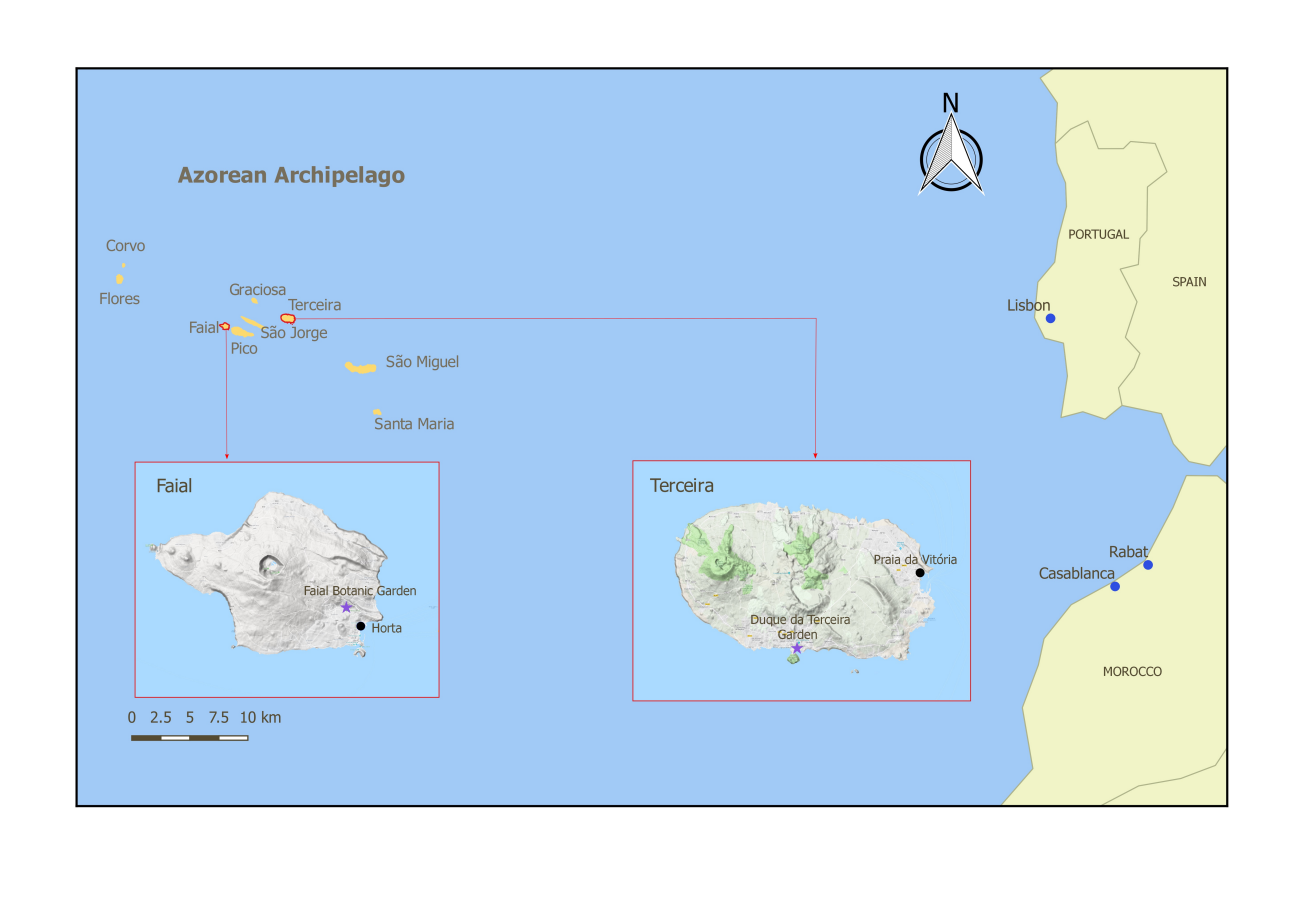
Map of the Azorean archipelago. The purple stars represent the location of Faial Botanic Garden and Duque da Terceira Garden on the islands of Faial and Terceira, respectively.

The Azores archipelago is composed of nine volcanic islands located in the northern Atlantic Ocean (Fig. 1). This archipelago has a temperate climate with mild average temperatures and high levels of humidity and rainfall that becomes more frequent and heavier in autumn and winter (Azevedo et al. 2004).

**Figure 2.**
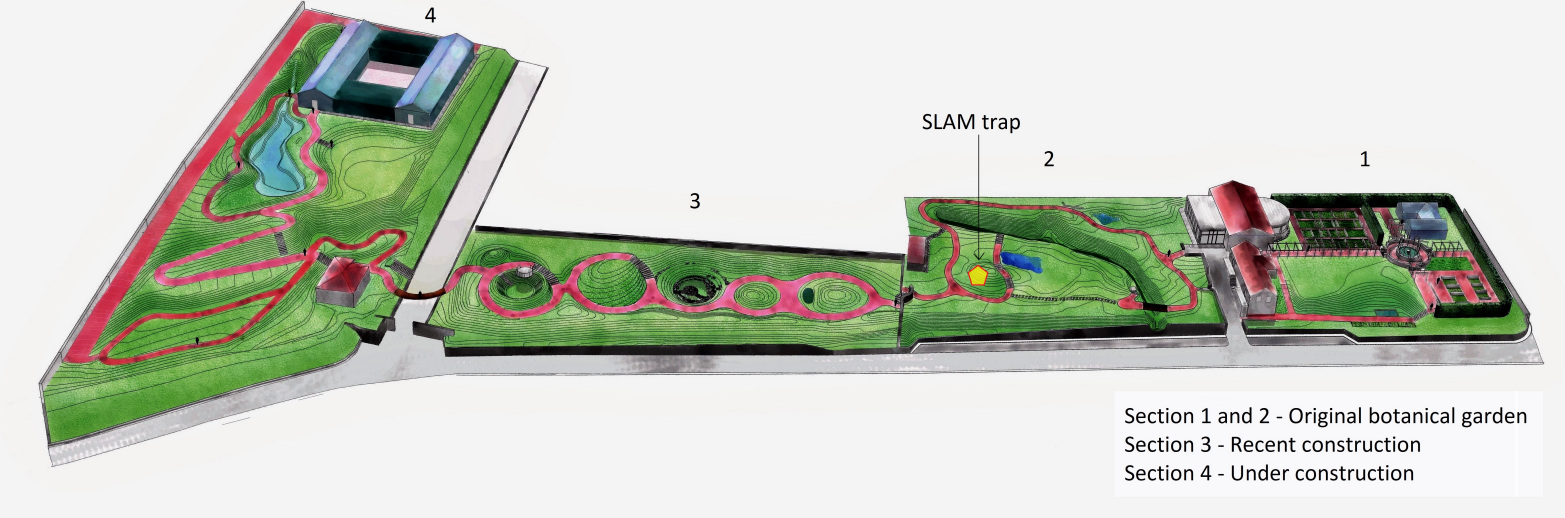
Faial Botanic Garden (“Jardim Botânico do Faial”). The garden is divided into four sections

**Figure 3.**
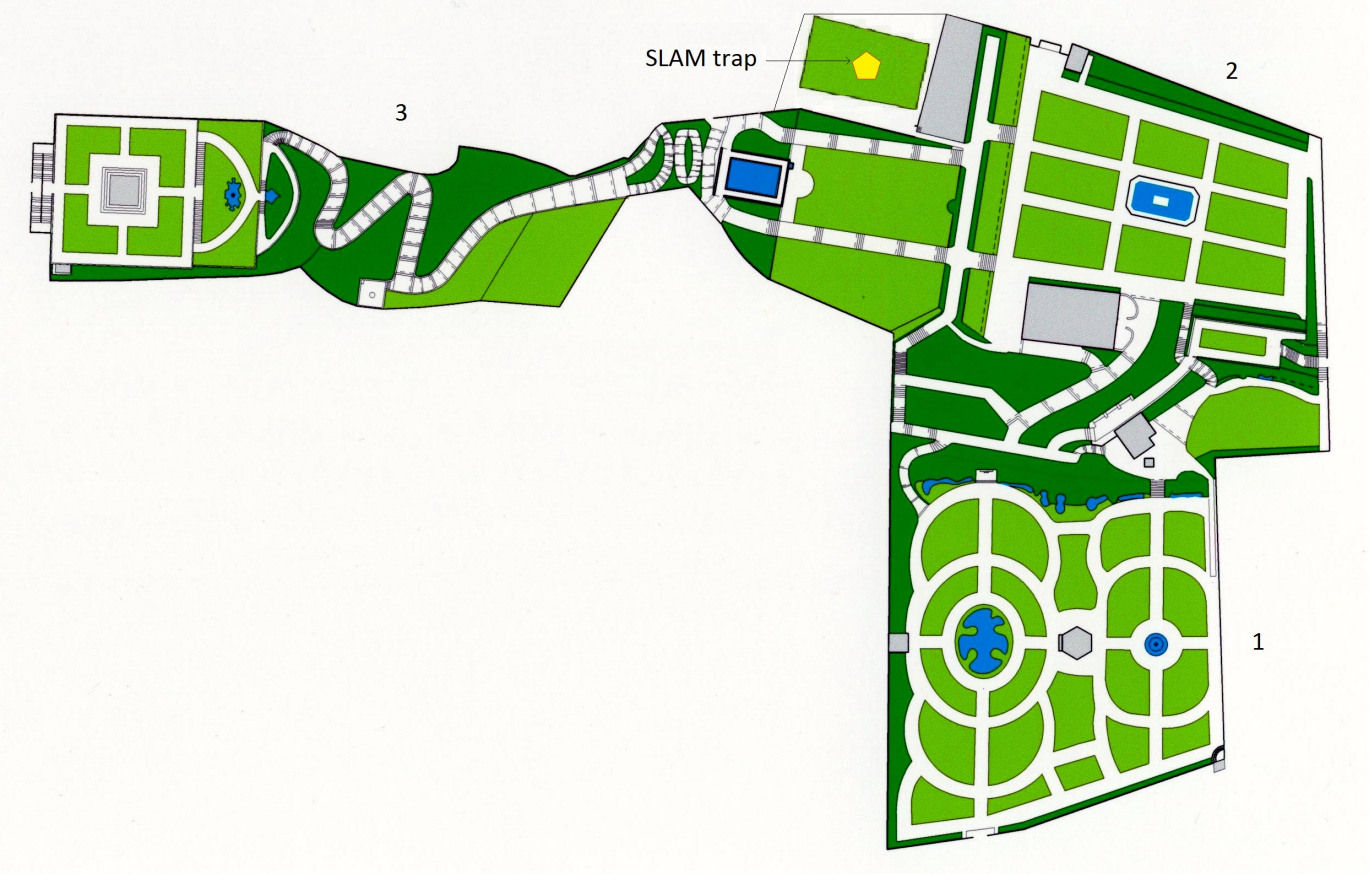
Duque da Terceira Garden (“Jardim Duque da Terceira”). The garden is divided into three sections according to their altitude, being 1 the lowest and 3 the highest.

We carried out our study in the gardens of Faial and Terceira Islands (Fig. 1). Faial Botanic Garden, «Jardim Botânico do Faial», is located in Horta (N 38°33'3.13", W 28°38'21.72") and it is divided into four sections (Fig. 2). This garden is composed of endemic and native vascular plant species from the Azores, including some exotic plant species common in the archipelago, especially those related to agriculture and medicinal purposes. Despite being located at low elevation (114 m), the high density of endemic plants in the old section of the garden (Fig. 2; section 2) creates a humid environment and conditions that simulate the native forests with a high cover of bryophytes and lichens in all substrates. The second garden, «Jardim Duque da Terceira», is situated in the centre of Angra do Heroísmo (N 38°39'9.10", W 27°13'8.44"). This public garden was created in 1882 and it is divided into three sections at different elevations, ranging from 29 m to 60 m above sea level (Fig. 3). Its design is different from Faial Botanic Garden, with a more sparse distribution of plants and a collection of exotic trees, shrubs and palms coming from all parts of the world (Anonymous 2020). The list of plant species for both gardens can be assessed in Suppl. material 1

### Arthropod sampling

**Figure 4.**
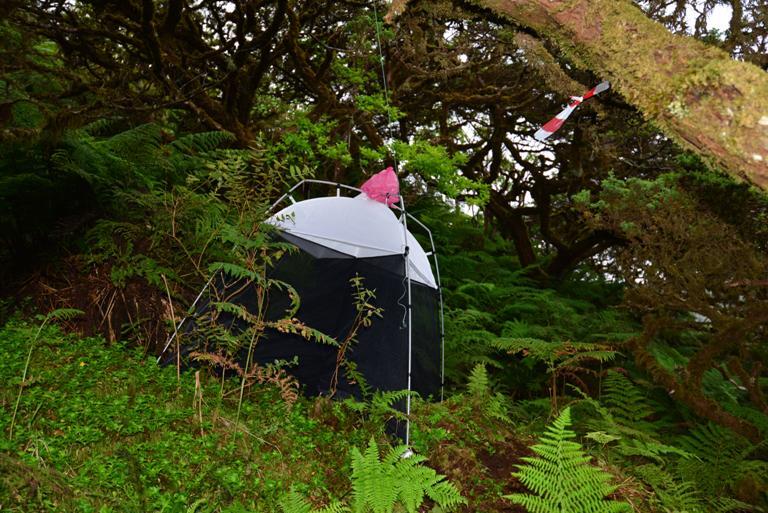
Passive flight interception traps termed *Sea, Land and Air Malaise* (SLAM) trap.

We used a combination of three different standardised techniques for sampling: nocturnal aerial active searching (AAS), nocturnal foliage beating (FBN) and passive flight interception traps termed *Sea, Land and Air Malaise* (SLAM) traps (Fig. 4). Both AAS and FBN are considered very adequate techniques to monitor spiders and beetles in native forests (Borges et al. 2018b) and are used to survey arthropods that are mostly active during the night.

We carried out active aerial searching for one hour at night at Duque da Terceira Garden in September 2017 and Faial Botanic Garden in June 2018. We sampled randomly in sections that contain plants species, representative of the communities at the garden. In Faial, we covered the first three sections of the garden (Fig. 2), whereas in Duque da Terceira Garden, we sampled mostly in section 2 (Fig. 3). We put every arthropod spotted above knee-level into vials with ethanol 96% for later identification. No data was retained on the arthropod species sampled per individual plant species.

We performed foliage beating at night (FBN) for one-night hour in both gardens, during the month of May 2018 in Terceira and in June 2018 in Faial covering a period of high arthropod activity. We beat tree and bush branches with the help of a wooden stick of approximately 1.5 m. We collected the fallen arthropods on a beating tray and we placed the specimens in ethanol 96%. No data were retained on the arthropod species sampled per individual plant species.

The SLAM traps were placed in representative sections of each garden (Figs 2, 3). In both gardens, the SLAM trap was set in section 2. The dimensions of the SLAM traps were roughly 110 cm × 110 cm × 110 cm (Fig. 4). Even though these traps were initially designed to target flying arthropods, it has been proved that other non-flying species can get trapped as they climb up the mesh or the trees from where the SLAM traps hang (Borges et al. 2017b, Matthews et al. 2019). The sampling recipients of the SLAM traps were set in April 2017 for both public gardens and checked monthly during six consecutive months until September 2017 (six samples for each garden). The plastic recipients contained propylene glycol (pure 1, 2-propanediol) for killing the arthropods,as well as for conserving the integrity of the specimens and their DNA material.

Overall, we collected ten samples in each garden, including six SLAM, three AAS and one FBN in Duque da Terceira Garden and six SLAM, two AAS and two FBN in Faial Botanic Garden.

These three sampling methods are complementary, as they allow the sampling of more active species with diurnal and nocturnal activity (SLAM) and species with nocturnal activity that are less mobile, plant specific or located in particular sections of the gardens (AAS and FBN). SLAM traps also collect non-flying species that climb up the mesh of the trap.

### Arthropod identification and data resources

First, we identified specimens at morphospecies level using a parataxonomy approach *sensu* Oliver and Beattie 1993 and then more precisely to species level. We only identified species from the following target taxa: Diplopoda, Chilopoda, Arachnida (excluding Acari) and Hexapoda (excluding Collembola, Lepidoptera, Diptera and Hymenoptera, but including Formicidae). We excluded these taxa as the morphospecies methodology is not appropriate for these arthropod groups due to the wide variation in their morphology. Two species of Coleoptera - *Scymnus interruptus* and *Scymnus nubilus* - were considered a single species (*Scymnus* sp.1), because their morphological differences are almost negligible and they could be easily misidentified. The colonisation status of the species was obtained from the checklist of Azorean arthropods (Borges et al. 2010). We classified each species as Azorean endemic, native (non-endemic Azorean species, but including Macaronesian endemics) and introduced in the Azores. We assumed that the taxa (morphospecies) that could not be identified or recognised (mostly from Duque da Terceira Garden) were “introduced”, since they had not been recorded on Terceira Island despite intensive research during the last 30 years.

All the samples were preserved in 96% ethanol, labelled, catalogued and stored in the University of the Azores arthropod collection “Dalberto Teixeira Pombo”. The data obtained from this study were included into a database for further statistical analyses (Suppl. material 2)

### Data analysis

#### Comparison of species richness and abundance

We first calculated sampling completeness to assess our collecting effort as our sample sizes amongst gardens differed considerably. We created rarefaction curves using the EstimateS programme v. 9.1.0 (Colwell 2020) and calculated sampling completeness as the ratio of the number of rarefied species out of the estimated number of species using the Jackknife 1 or Chao 1 non-parametric estimates, depending on which one showed a smoother curve (see also Hortal et al. 2006). In order to evaluate how well these estimators performed, we calculated the final slopes of the estimated species accumulation curves as suggested by Cardoso et al. 2009. We repeated this evaluation for the total (adults and immature) and adult arthropod communities of both gardens as well as for each studied order (Araneae, Coleoptera and Hemiptera).

We calculated a series of diversity measures: species abundance (N), species richness estimates (S), the number of singletons and doubletons and the second and third Hill series numbers, i.e. Shannon-Wiener exponential index (exp H’) and inverse Simpson’s index (1/D) (Jost 2006). We also calculated the inverse of Berger-Parker index (1/d), which represents the proportional abundance of the most common species in the population (Berger and Parker 1970). The greater the value of 1/d, the more diverse is the habitat (Magurran 2004). This is a dimensionless number and thus, more robust when comparing areas with different sample sizes (Caruso et al. 2006). We tested the differences iBerger-Parker index between the two communities through a randomisation test with 10,000 random partitions (Solow 1993) using Species Diversity and Richness software (Seaby and Henderson 2007).

We also calculated the following measures for endemic, native non-endemic and introduced species of each garden separately: the species number, the number of adults and individuals and the abundance, colonisation and trophic status of the most dominant species. The same procedure was followed when analysing the orders Araneae, Coleoptera and Hemiptera separately. For the order Coleoptera, no juveniles were found in the samples and, therefore, calculations were done only with adult individuals. Five morphospecies, previously collected in Azores in other projects, were not considered in the analysis because of their undetermined colonisation status, including three Coleoptera (eight adults) and two arachnids (22 individuals, 12 of those adults)

#### Comparison of species composition

We compared the composition of both communities using β‐diversity (β_Tot_) and its replacement/turnover (β_Repl_) and richness difference (β_Rich_) components (Carvalho et al. 2012), in which:

β_Tot_ = β_Rep_ + β_Rich_

β_Rep_ = 2 * ((min(*b*, *c*)) / (*a* + *b* + *c*)

β_Rich_ = [*b* - *c*] / (*a* + *b* + *c*)

where *a*, is the number of species common to both sites, *b* the number of species exclusive to the first site and *c* the number of species exclusive to the second site; min(*b*, *c*) is the minimum number of exclusive species. This quantity is multiplied by two because each substitution involved two different species (Carvalho et al. 2012). [*b* - *c*] is the absolute difference between the number of exclusive species in both sites.

We calculated beta diversity using incidence and abundance data with the Jaccard dissimilarity index. The incidence data indicate changes in the number and identity of the species while the abundance data are sensitive to changes in the distribution of the individuals belonging to different species. We performed calculations with the total number of individuals sampled in each garden and separately for the adult specimens of Araneae, Coleoptera and Hemiptera. We used the package BAT (Cardoso et al. 2020) of the R software.

#### Species dominance patterns

In order to estimate species dominance patterns, we ranked the species according to their abundance in our samples. We created different lists for each garden, with data based on adult and total sampled community. We identified the most common species that represented at least half of the individuals in the community (≥ 50%) (see Hubbell 2013) and we determined their colonisation status: endemic, native and introduced, as well as their trophic status: predator, herbivore and saprophage. The same classification was performed for each of the studied orders: Araneae, Coleoptera and Hemiptera.

## Results

In this study, we collected a total of 8356 arthropod specimens. Duque da Terceira Garden had more arthropods than Faial Botanic Garden, with 4563 and 3793 individuals, respectively (Table 1). Chao1 estimator had the most stabilised curve and thus it was used to calculate sampling completeness of both botanic gardens. Values of completeness were above 0.7. In addition to this, all the slope values for the species accumulation curves were lower than 0.01, suggesting that inventory completeness was nearly achieved.

### Comparison of species richness and abundance

The number of observed species was 88 for Faial and 191 for Terceira, corresponding to 85% and 73% of the estimated richness, respectively. When comparing species richness using adult data, the number of rarefied species for Duque da Terceira Garden was higher than for Faial Botanic Garden (114 versus 79 species) and when using all specimen data (172 versus 88 species). The Shannon-Wiener exponential index (exp H’), inverse Simpson’s index (1/D) and inverse Berger-Parker index (1/d) showed that the botanic garden of Terceira was more diverse when using both adult (exp H’ = 29.24; 1/D = 14.45; 1/d = 6.5) and total individual data (exp H’ = 35.94; 1/D = 17.97; 1/d = 6.84) (Table 2). The difference in the values of the inverse Berger-Parker index between gardens was statistically significant at 5% level (Table 2).

Duque da Terceira Garden also held the largest proportion of rare species, nearly 47% of the total species number. In this garden, singletons were almost three times more abundant than in Faial Botanic Garden (55.67 versus 19) and doubletons were twice as many as those found in Faial (24.83 versus 12). These rare species are predominantly introduced species and some of them still need proper taxonomic identification (see Suppl. material 2).

### Patterns in endemic, native and introduced species

**All species**

Regarding exotic species, Terceira garden showed a greater number of species: 135 species with 2219 individuals. These figures almost tripled the number of introduced species in Faial garden with 49 species and 777 individuals. Differences were more pronounced when considering only adult individuals (Table 3). Conversely, Faial Botanic Garden had two more endemic species than Terceira and three times more specimens of endemics (Table 3). Regarding native species, Duque da Terceira Garden held 15 more species than Faial, but with lower total abundance, this community being mostly formed by adult specimens. The situation was inverse for Faial with juveniles contributing in a large proportion to the total number of native species (Table 3).

In terms of dominance, our data showed that the most common species were either native (three species) or endemic (one species) and no introduced species dominated (Table 4). In Terceira, the adult and total sampled communities were dominated by the native *Trichopsocus clarus* (Banks, 1908) (Psocoptera), whereas in Faial Botanic Garden the endemic Psocoptera, *Cerobasis* sp.1 was the most common species amongst adult specimens (27%). The native Hemiptera, *Cyphopterum adcendens* (Herrich-Schäffer, 1835) dominated the total sampled community of Faial (19%).

**Araneae**

Sampling completeness for this order was achieved as reported by the Jacknnife1 estimator, which was above 65% and 70% for the adult and the total number of individuals; and by the low values of the species accumulation curve slopes (< 0.08) (Table 5). The garden of Terceira Island harboured a higher number of rarefied species than Faial’s garden, but in lower abundance (Table 5). All diversity indices and the statistical comparison for the 1/d index, registered a greater diversity of spiders in Faial for the adult and the total community (P < 0.05). The only exception was the Shannon-Wiener exponential value obtained for the adult data in Terceira, which was slightly higher for this garden (Table 6). Around 50% of the registered species were singletons or doubletons, with Terceira data having a slightly greater number than Faial (Table 6).

Duque da Terceira Garden was richer in introduced and native species (Table 7). However, the total abundance of introduced species in Faial was 3.5 times larger than in Terceira. Only one endemic spider species was found in both gardens, *Emblyna acoreensis* Wunderlich, 1992 which was the most common species in the adult community of Faial Botanic Garden (Table 8). Conversely, the total sample community was dominated by an introduced spider species, *Neoscona crucifera* (Lucas, 1838) (39%) (Table 8). The native species *Porrhoclubiona decora* (Blackwall, 1859) dominated the adult and the total spider community in Duque da Terceira Garden, in proportions of 38% and 59%, respectively.

**Coleoptera**

Sample sizes differed widely between gardens (Table 5). In Faial, we sampled 178 specimens, while in Terceira, we counted 1465 individuals. Despite this striking difference, the completeness values using the Jackknife1 estimator were high for both inventories, with the final slope values of the species accumulation curves close to zero. Even with a larger sample size, Terceira held nearly the same number of rarefied species as Faial (Table 5). Diversity indices registered higher diversity in Faial Botanic Garden, although the Inverse Berger-Parker index was lower in this garden (p < 0.05) indicating that the most abundant species had a higher dominance (Table 6). In terms of singletons and doubletons, Terceira held a high proportion of rare species, 65% of all sampled species, when compared with Faial (43%) (Table 6). The number of introduced beetles dominated on a larger scale in the botanic garden of Terceira compared to Faial (Table 7). Two introduced species, both predatory beetles, were the most abundant Coleoptera in both gardens: *Tachyporus nitidulus* (Fabricius, 1781) in Faial (27%) and *Sericoderus lateralis* (Gyllenhal, 1827) (18%) in Terceira (Table 8). The garden of Terceira harboured more native species with more individuals than Faial, but only one endemic species, *Heteroderes azoricus* (Tarnier, 1860), was found in this garden. The same species was also present in the botanic garden of Faial, with another endemic species, the Curculionidae *Calacalles subcarinatus* (Israelson, 1984).

**Hemiptera**

Inventory completeness, estimated with Jackknife1, was achieved for the four groups of Hemiptera, with ratios not lower than 0.7 and slope values of the estimators’ curves not larger than 0.07 (Table 5). The total abundance of Hemiptera in Faial was twice as high as that obtained for Terceira, with juveniles dominating (Table 5). Duque da Terceira Garden accounted for a larger number of rarefied species in the total and the adult sampled community (Table 5). All diversity indices showed that Terceira garden is more diverse, with the exemption of the 1/d index for the adult individuals, but this difference was not significant at 5% level (Table 6). The percentage of rare species was approximately 30% in both gardens, but the proportion of singletons and doubletons in Terceira for the adult sample doubled the values obtained in Faial (Table 6).

Duque da Terceira Garden showed a richer number of introduced species and with larger communities (Table 7). This garden had a higher number of native species too, but the overall abundance in Faial was ten times larger than in Terceira. In both gardens, the native species *Cyphopterum adcendens* dominated nearly half of the total sampled community (Table 8). Another native species, *Loricula elegantula* (Bärensprung, 1858) was the most common in Faial if considering only adults, whereas in Terceira, it was the introduced herbivore *Oxycarenus lavaterae* (Fabricius, 1787) (Table 8). Endemic species were more common in Faial (three species), while only a single species belonging to the family Aleyroridae was sampled in Terceira.

### Comparison of species composition

**All species**

**Figure 5.**
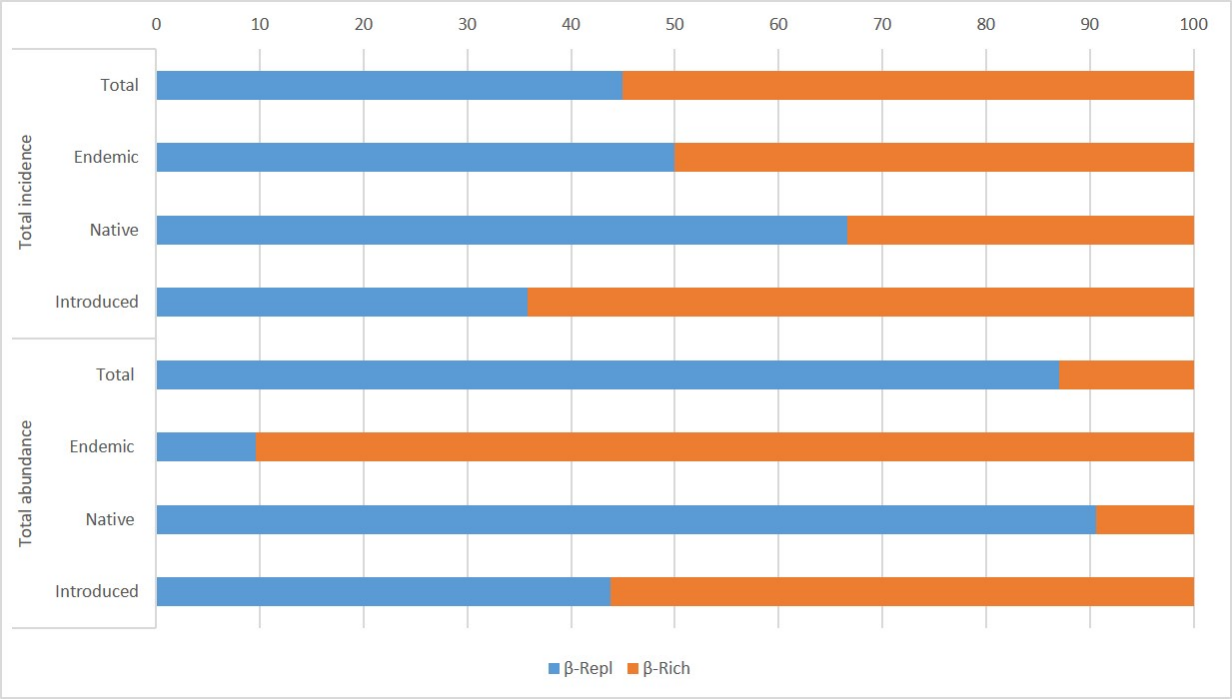
Total beta diversity partition (β_Tot_) using its replacement/turnover (β_Repl_) and richness difference (β_Rich_) components for all arthropods between Faial Botanic Garden and Duque da Terceira Garden.

The total beta diversity (β_Tot_) of all arthropods between the two botanic gardens was 0.8 for incidence data and 0.83 for abundance data, with beta richness (β_Rich_) representing 55% of the former and beta replacement (β_Repl_) 87% of the latter (Fig. 5). Regarding endemic species, β_Rich_ and β_Repl_ contributed equally to the variation in beta diversity for the incidence data, while, for the abundance data, β_Rich_ accounted for 90%. For the native species, β_Repl_ represented most of the beta diversity, whereas β_Rich_ contributed to most of the total beta diversity of introduced species.

**Araneae**

**Figure 6.**
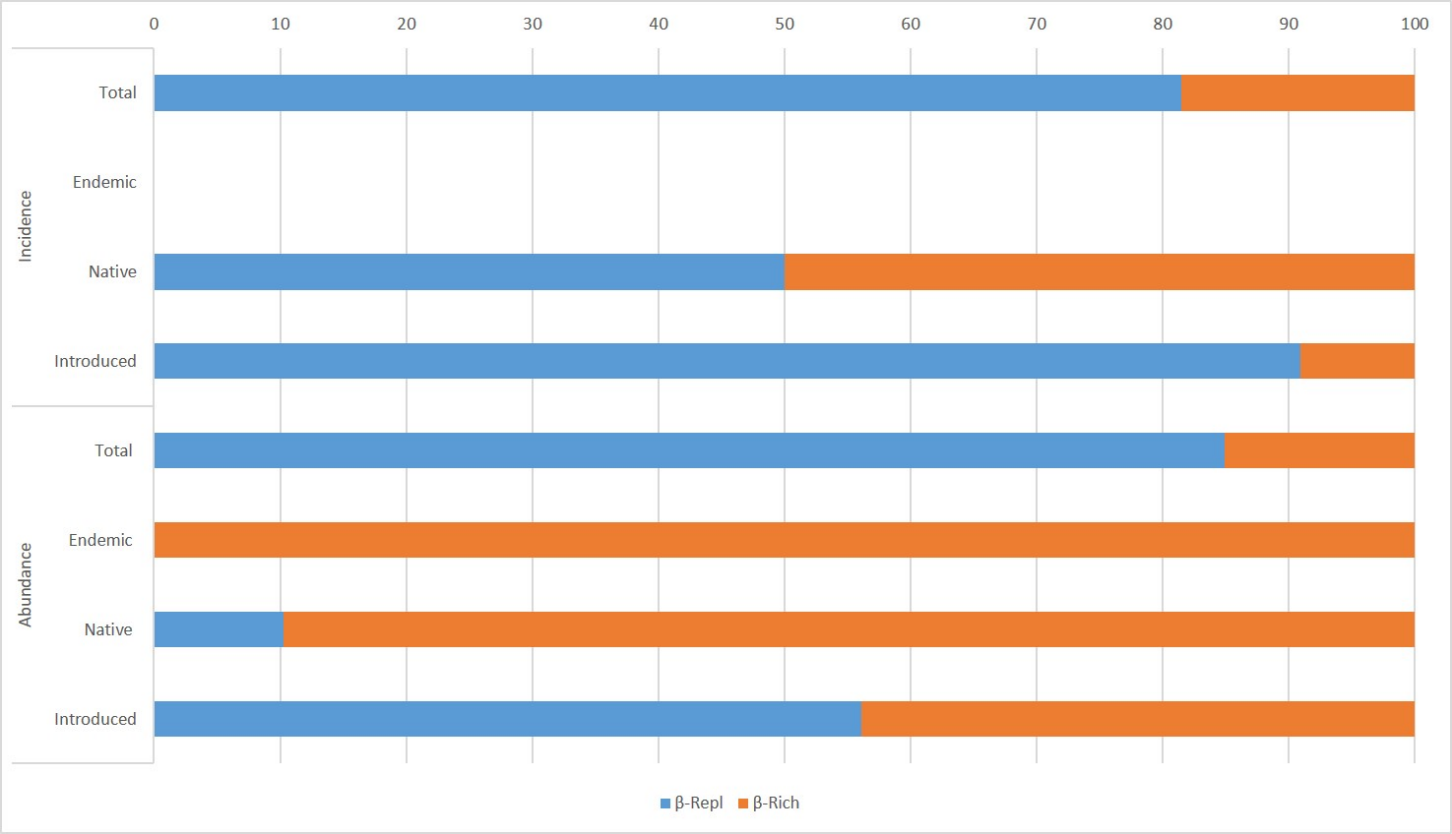
Total beta diversity partition (β_Tot_) using its replacement/turnover (β_Repl_) and richness difference (β_Rich_) components for adult Araneae specimens between Faial Botanic Garden and Duque da Terceira Garden.

The total beta diversity of the incidence of spiders was 0.75, which was caused mainly by β_Repl_ (81%) (Fig. 6). For the abundance data, β_Tot_ was 0.78, of which β_Repl_ accounted for 88%. Both gardens shared a unique endemic species, *Emblyna acoreensis* and thus, β_Rich_ and β_Repl_ were zero for incidence data. For abundance data, β_Rich_ accounted for 100% of the β_Tot._ For native species, 90% of β_Tot_ was accounted for by β_Rich_, whereas, for incidence data, β_Repl_ and β_Rich_ contributed equally (50%). Regarding introduced species, β_Repl_ accounted for 91% of the β_Tot_ of incidence and for 56% of abundance.

**Coleoptera**

**Figure 7.**
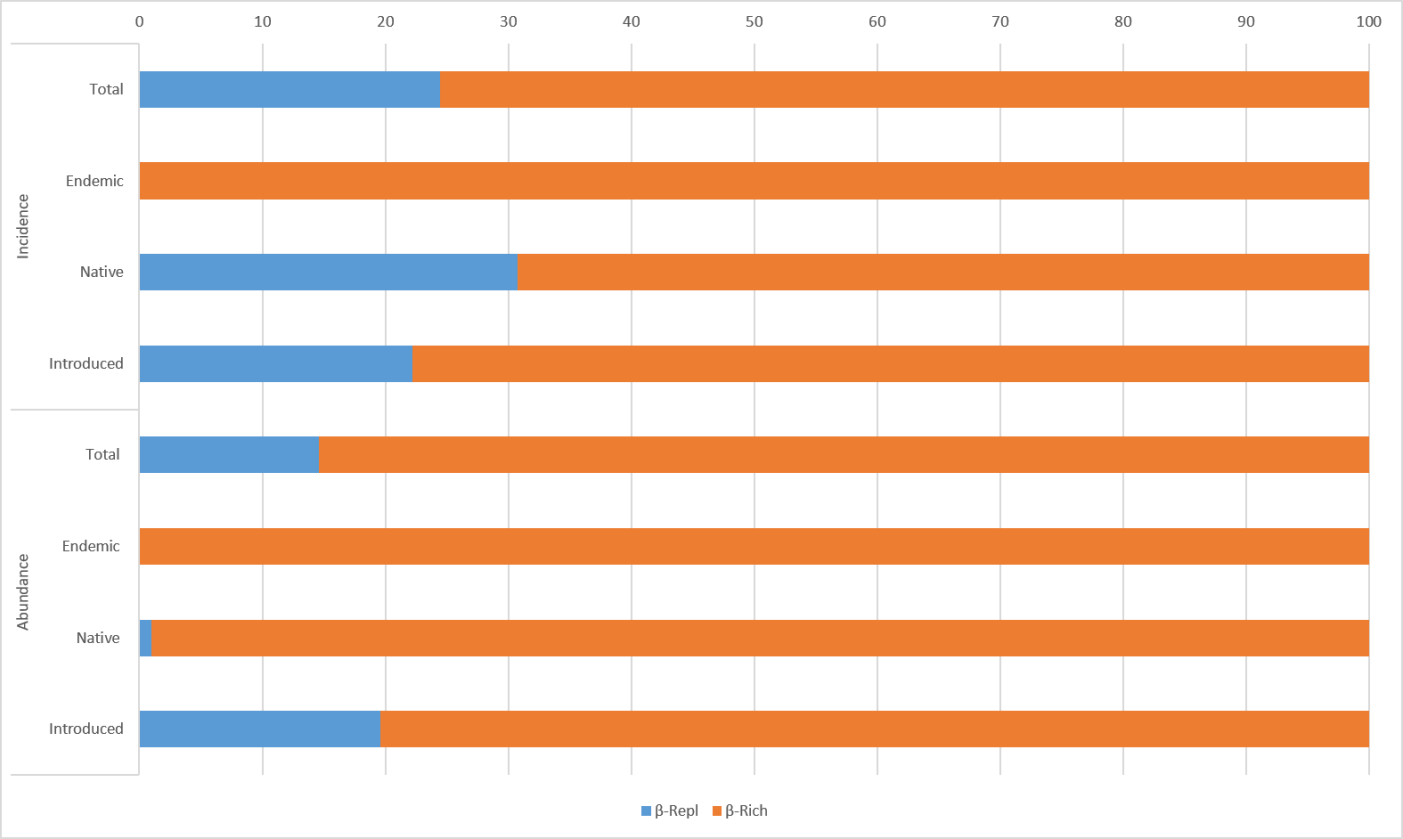
Total beta diversity partition (β_Tot_) using its replacement/turnover (β_Repl_) and richness difference (β_Rich_) components for adult Coleoptera specimens between Faial Botanic Garden and Duque da Terceira Garden.

Incidence and abundance data of beetles indicated that β_Rich_ contributed 76% and 85% to β_Tot_, respectively (Fig. 7). This pattern was the same when analysing the endemic, native and introduced species of Coleoptera separately, where β_Rich_ explained between the 80% and 100% of the β_Tot_ of both incidence and abundance.

**Hemiptera**

**Figure 8.**
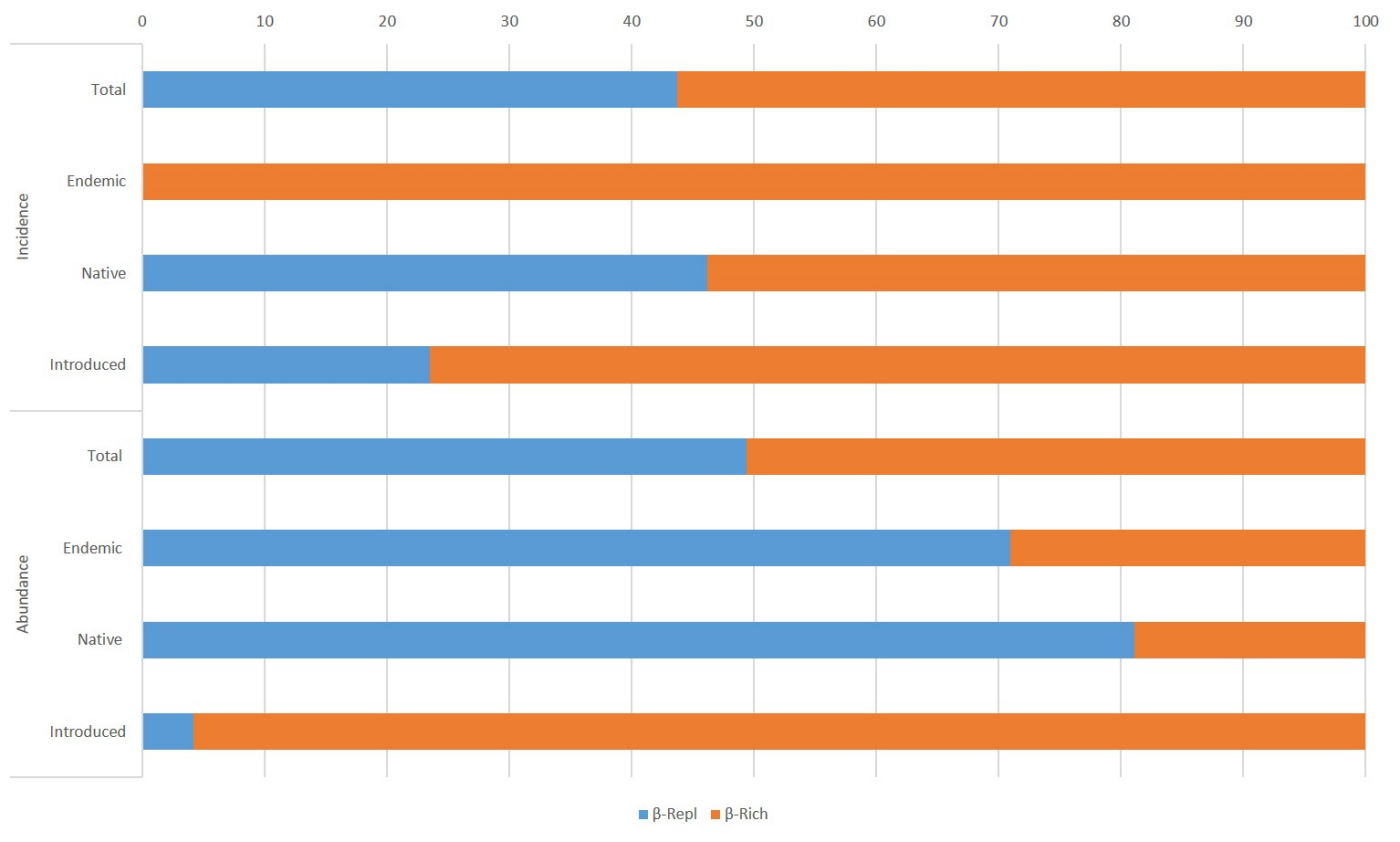
Total beta diversity partition (β_Tot_) using its replacement/turnover (β_Repl_) and richness difference (β_Rich_) components for adult Hemiptera specimens between Faial Botanic Garden and Duque da Terceira Garden.

As for the Hemiptera, the β_Tot_ was 0.82 for incidence data and 0.94 for abundance data, with β_Rich_ and β_Repl_ contributing with approximately 50% in both cases (Fig. 8).

Total beta diversity of the incidence of endemic species was completely explained by β_Rich_ (100%), while β_Repl_ contributed more to the differences (71%) in their abundance. Total beta diversity of the abundance and incidence of native species was driven mostly by β_Repl_ (81%) and by β_Rich_ (54%), respectively. Both incidence and abundance data for introduced species indicated that β_Rich_ explained most of the β_Tot_ between Faial and Terceira gardens (76% incidence and 96% abundance data).

### Species dominance patterns

In Faial Botanic Garden, two species of Psocoptera (*Cerobasis* sp.1 and *Trichopsocus clarus*) dominated the adult sampled community, covering almost 40%. They were followed by a native species of Opiliones, *Leiobunum blackwalli* Meade, 1861 (9%) and by the native ant *Lasius grandis* Forel, 1909 (7%). When considering the total sampled community, the Hemiptera species, *Cyphopterum adcendens* (19%) led the list, followed by the two Psocoptera species *Trichopsocus clarus* and *Cerobasis* sp.1, with 13% and 11%, respectively. The most common species in Faial were either endemic or native to Azores (Suppl. material 3). In Terceira, the Psocoptera *Trichopsocus clarus* (15%) and the Formicidae *Lasius grandis* (12%) dominated amongst adult specimens. Even when pooling together data from juveniles and adults, these two species continued to dominate, accounting for one quarter of the collected specimens in Terceira. Both species are native to the Azores (Suppl. material 3).

Regarding spiders, the most abundant species amongst the adult specimens of Faial were the endemic *Emblyna acoreensis* and the introduced species *Entelecara schmitzi* Kulczynski, 1905, both in equal proportion (20%). When considering all specimens, another introduced species, *Neoscona crucifera*, led the list, accounting for 39%, due to its abundant juvenile population. This species nearly doubled the number of individuals of the native species *Porrhoclubiona decora*, which was the most dominant species amongst the adult and all specimens of spiders in Terceira, with 38% and 59%, respectively (Suppl. material 4). In Faial, the most common Coleoptera species, that represented 50% of adults, were all introduced: *Tachyporus nitidulus* (27%), *Longitarsus kutscherae* (Rye, 1872) (14%) and a species of the family *Chrysomelidae* (11%). In Terceira, other two introduced beetles led the list, *Sericoderus lateralis* (18%) and *Epitrix cucumeris* (Harris, 1851) (12%) (Suppl. material 4).

For the Hemiptera community, in Faial, the native *Loricula elegantula* and the endemic species *Strophingia harteni* Hodkinson, 1981 were the most common species, accounting for 31% and 26% of adult specimens, respectively (Suppl. material 4). More than half of all collected specimens in this garden belonged to the native species *Cyphopterum adcendens*, due to its high number of juveniles. Conversely, the introduced species *Oxycarenus lavaterae* was dominant in Terceira for both the adult (34%) and the total number of individuals (41%) (Suppl. material 4).

## Discussion

Urbanisation and development of large cities in rural areas implies the loss of native biodiversity in most cases (McKinney 2006). Therefore, the creation of public and private gardens that enhance native wildlife is of great importance in biodiversity conservation (Sattler et al. 2010). These gardens can act not only as reservoirs of native and endemic species, but also as connections amongst natural species populations (Roberts et al. 2007). How gardens are designed in terms of species identity and richness will determine, to a great extent, the arthropod community living in them. In our study, we carefully selected two historic gardens with plant communities of different biogeographic features: Faial Botanic Garden with mostly native and endemic plant species and Duque da Terceira Garden dominated by exotic plant species.

### Comparison of species richness and abundance

As hypothesised, in Faial garden, where indigenous plants dominated, endemic and native arthropods were better represented, with native species populations being the most abundant. However, the number of exotic arthropod species (49) was still greater than the native and endemic species together (39). This may be a consequence of two conditions: i) the location of the botanic garden of Faial, surrounded by agricultural fields with a continuous source of new introductions of exotic arthropods; and ii) the fact that the current nearest source of native vegetation is at 900 m of elevation and at a long distance, becoming geographically isolated from natural populations.

Our second hypothesis was also confirmed, the richness and abundance of species richness of introduced species was higher at Duque da Terceira Garden. In addition, we observed that Duque da Terceira Garden has higher levels of overall arthropod diversity. This was mainly due to the high number of rare species, 55 singletons and 24 doubletons, especially within the order Coleoptera. Of particular relevance is the fact that the most abundant Coleoptera species in both Terceira and also Faial, were introduced. This can be the result of either the existence of an easy entrance pathway (Méquignon 1935) or their great dispersal ability (Borges et al. 2013).

Human activities, including commercial trade, horticulture and ornamental gardening, could help explain our findings (Borges et al. 2013). In Azores, a large fraction of arthropods is considered introduced (Borges et al. 2010, Rego et al. 2015). Roques et al. 2009 reported a large number of invertebrate introductions on islands, many of them released for biological control. However, a large fraction of those species are introduced through the horticultural and ornamental trade. It is known that Duque da Terceira Garden was used as a “nursery acclimation garden” for tropical plants before their potential admittance in Europe. Therefore, it is not surprising to find high numbers of exotic arthropods in this garden (see also Pérez Santa-Rita et al. 2018). This pathway of exotic insect entrance in Azores was recognised by Méquignon 1935) as an explanation for an important number of exotic beetles with American origin occurring in the Azores. Future analysis of the worldwide distribution patterns of the introduced (morpho)species may shed some light on whether transcontinental introductions are common.

### Comparison of species composition

The community composition of both botanic gardens differed as was confirmed by the high value of the total beta diversity of all arthropods between gardens. Duque da Terceira Garden contained larger numbers of introduced species of Hemiptera and Coleoptera and, hence, beta richness accounted for most of the total beta diversity of exotic species between botanic gardens. The exotic *Oxycarenus lavaterae* was, by far, the dominating species (41% of the total number of sampled specimens). One possible explanation for the absence of *Oxycarenus lavaterae* in Faial Island could be the restriction in food sources, as this species feeds on Malvaceae plants that are common in only Duque da Terceira Garden. This species, originally from west-Mediterranean areas, has rapidly spread across Europe and, nowadays, it is considered a potential future pest (Nedved et al. 2014). In the case of Faial Botanic Garden, the native species *Cyphopterum adcendens* formed 54% of the total community. The high diversity of native species in Faial Garden is the main reason why beta replacement explains 81% of the total beta diversity of native species between both botanical gardens. The international garden seed exchange can have a great effect on the spread of exotic species, counting for part of the so-called accidental successful invasions (Lowe et al. 2000). It is now widely recognised that the horticulture use by nurseries and botanical gardens is one of the most important pathways for the entrance of exotic plant species to new geographic areas (Reichard and White 2001). Those exotic plants may host other organisms like pathogens and insects that, if adapted to the new environment, can spread. The contribution of botanic gardens to the accidental introductions of exotic arthropods in the Azorean archipelago is unknown, but our results point in this direction.

Our study reported seven new species records for the island of Faial and five additional for Terceira Island, with three and four, respectively, being exotic species (Suppl. material 2). Two species were recorded for the first time in the Azores archipelago. In Terceira, we found one specimen of the exotic beetle *Sirocalodes mixtus*, which had been previously reported on the Portugal mainland (Campobasso et al. 1999), but not in the Azores. This species has spread across southern and western Europe along with their host plants from the family Papaveraceae, *Ceratocapnos claviculata* and *Fumaria officinalis* (Germann 2013), a family scarcely represented in both gardens. In Duque da Terceira Garden, we collected fifteen individuals of the endemic Macaronesian spider *Paidiscura orotavensis*, which had already been recorded in the Madeira archipelago and the Canary Islands (Crespo et al. 2014; Malumbres-Olarte et al. 2020), but not in the Azores (cf. Borges et al. 2010).

The creation of green spaces embedded in urban matrices not always implies positive conservation outcomes, as the capacity of native species to establish in other habitats not only depends on the ecological characteristics of the new habitat, but also on the ability of the species to disperse and adapt (Fattorini et al. 2017). Therefore, conservation strategies should be based on species-specific cases when possible. For instance, the spider community in Duque da Terceira Garden was mostly represented by the native species *Porrhoclubiona decora* (59%), leading to abundance beta richness accounting for almost all of the total beta diversity within native species. Even though the dispersal ability of *Porrhoclubiona decora* is low, its great abundance suggests that its ability to adapt to novel plants and compete is remarkable. Interestingly, the situation is the opposite in Faial Botanic Garden, where the native *Porrhoclubiona decora* was outnumbered by the exotic species *Neoscona crucifera*. Habitat complexity is important for spider communities (Malumbres-Olarte et al. 2012) and, consequently, the 3D structure of exotic plant species in Duque da Terceira Garden might favour *Porrhoclubiona decora* dominance. This result is in line with Bezemer et al. 2014 who demonstrated that exotic plants might open novel habitats that provide ecosystem services to native and endemic arthropod species capable of shifting hosts. However, exotic plants could also favour the establishment of new introduced species, which can explain the great turnover of exotic species among gardens.

### Conservation value of the studied gardens

Interestingly, no introduced species had a dominant role in any garden, despite being part of the 50% most abundant species in Terceira. Notably, in Faial Botanic Garden, the 50% most abundant species are either endemic or native non-endemic. This repository of indigenous fauna is of conservation value and has the potential for naturalisation projects for the area. As has been suggested, urban green spaces cannot replace pristine habitats. However, they have the potential to enhance native diversity and act as species corridors between natural habitats (Kowarik 2011). This could be the case of Faial Botanic Garden, which holds a large community of native species and, thus, it has the potential to be part of corridors of native plants across the agricultural landscape.

## Conclusions

With some few exceptions for some native insect species that are more abundant in the Duque da Terceira Garden, the two initial hypotheses were mostly confirmed. Indeed, the colonisation status of plant communities influenced the arthropod species sampled in each garden: i) the richness and abundance of endemic and native non-endemic arthropods was higher at the Faial Botanic Garden and (ii) the richness and abundance of exotic arthropods was higher at the Duque da Terceira Garden. This study improves our knowledge about urban arthropod diversity in the Azores and shows how well-designed urban garden management and planning contribute to the conservation of native and endemic Azorean species.

Future research should focus on determining whether native species in Faial can establish self-sustaining populations and survive in the long-term, despite being a semi-natural habitat. Long term monitoring in Duque da Terceira Garden is also vital to detect changes in the population size and distribution of introduced arthropod species. It would be also desirable to have more information on the plants of each garden (for example, percentage of native, endemic, introduced, geographic/taxonomic origin), as well as to sample each plant individually to investigate insect-plant specific interactions. The surrounding vegetation is also important to understand if the role of these gardens acts as sink or source populations.

In addition, a well-designed management plan and practices would be beneficial at both historical urban gardens that were recently under construction for expansion, with the creation of new habitats. This study highlights the importance of urban parks with well-planned strategies and appropriate management policies aimed to reduce threats to native species and to increase their conservation.

## Supplementary Material

2F268769-C7ED-5766-B975-F73BDDC6EFB5 10.3897/BDJ.8.e54749.suppl1 Supplementary material 1 List of plant species of Faial Botanic Garden (JBF) and Duque da Terceira Garden (JDT) Data type Occurrences Brief description The complete list of Vascular Plants of Faial Botanic Garden (JBF) and Duque da Terceira Garden (JDT) File: oo_412495.xlsx https://binary.pensoft.net/file/412495 Gabriel, R. & Casimiro, P.

39205493-FEDB-5DCF-8E8B-40FCB5F47EBC 10.3897/BDJ.8.e54749.suppl2 Supplementary material 2 Arthropod species list of Faial Botanic Garden and Duque da Terceira Garden Data type Occurrences Brief description The distribitiona and abundance of species in Faial Botanic Garden and Duque da Terceira Garden File: oo_412546.xlsx https://binary.pensoft.net/file/412546 Borges, P.A.V.

37A0EF1F-B0F3-5E96-AE71-7E5263207F0E 10.3897/BDJ.8.e54749.suppl3 Supplementary material 3 The most common species in Faial Botanic Garden (FAI) and Duque da Terceira Garden (TER) - ALL SPECIES Data type Abundance Brief description The most common species in Faial Botanic Garden (FAI) and Duque da Terceira Garden (TER) that represented half or more of the individuals in the community (≥ 50%). Colonisation status (E- endemic, N – native, I – introduced) and trophic status (P – predators; H – herbivores; S – saprophages) File: oo_412603.xlsx https://binary.pensoft.net/file/412603 Arteaga, A. & Borges, P.A.V.

C4403826-8830-536F-A6FE-94F690B7F7A4 10.3897/BDJ.8.e54749.suppl4 Supplementary material 4 The most common species in Faial Botanic Garden (FAI) and Duque da Terceira Garden (TER) that represented half or more of the individuals in the Araneae, Coleoptera and Hemiptera community (≥ 50%). Data type Abundance Brief description The most common species in Faial Botanic Garden (FAI) and Duque da Terceira Garden (TER) that represented half or more of the individuals in the Araneae, Coleoptera and Hemiptera community (≥ 50%). colonisation status (E- endemic, N – native, I – introduced) and trophic status (P – predators; H – herbivores; S – saprophages) File: oo_412602.xlsx https://binary.pensoft.net/file/412602 Arteaga, A. & Borges, P.A.V.

## Figures and Tables

**Table 1.** Abundance (N), observed species richness (S), rarefied species richness for Terceira, based on the same individuals of Faial, final slopes of the estimated species accumulation curves and completeness, based in two methods (see methods) for Faial Botanic Garden (FAI) and Duque da Terceira Garden (TER). (*) indicates higher level of biodiversity, based on the rarefaction method.

		**N**	**S (observed)**	**S (rarefied)**	**Slope**	**Completeness**	
						**Jackknife1**	**Chao1**
**Adults**	**FAI**	1505	79	79	0.007	0.77	0.78
	**TER**	3773	184	113.83*	0.003	0.69	0.71
**Total**	**FAI**	3793	88	88	0.003	0.78	0.85
	**TER**	4563	191	172*	0.002	0.70	0.73

**Table 2.** Number of rare species (singletons and doubletons), percentage of rare species, based on data from Table 1 and Hill diversity metrics for Faial Botanic Garden (FAI) and Duque da Terceira Garden (TER): H´ - Shannon-Wiener index; D – Simpson index; d – Berger-Parker index. For Berger-Parker index, (*) indicates higher level of biodiversity, based on the randomisation test with 10,000 random partitions (Solow 1993).

		**Singletons**	**Doubletons**	**Rare sp** %	**Hill series**		
					**exp H**'	**1/D**	**1/d**
**Adults**	**FAI**	20	9	36.70	20.64	9.22	3.62
	**TER**	40.23	17.76	50.90	29.24	14.45	6.5*
**Total**	**FAI**	19	12	35.22	19.98	11.27	5.23
	**TER**	55.67	24.83	46.8	35.94	17.97	6.84*

**Table 3.** Number of endemic, native and introduced species (S) and individuals (N) in Faial Botanic Garden (FAI) and Duque da Terceira Garden (TER).

		**Endemic**		**Native**		**Introduced**	
		**N**	**S**	**N**	**S**	**N**	**S**
**Adults**	**FAI**	522	9	667	30	316	49
	**TER**	183	7	1773	45	1797	135
**Total**	**FAI**	618	9	2398	30	777	49
	**TER**	187	7	2127	45	2219	135

**Table 4.** Dominant species in Faial Botanic Garden (FAI) and Duque da Terceira Garden (TER). Colonisation status (E- endemic, N – native) and trophic status (S – saprophages; H – herbivores).

		**Dominant species**	**Order**	**Colonisation status**	**Trophic status**	**Abundance**	**N_total_**
**Adults**	**FAI**	*Cerobasis* sp. 1	Psocoptera	E	S	415 (27%)	1505
	**TER**	*Trichopsocus clarus*	Psocoptera	N	S	579 (15%)	3753
**Total**	**FAI**	*Cyphopterum adcendens*	Hemiptera	N	H	725 (19%)	3793
	**TER**	*Trichopsocus clarus*	Psocoptera	N	S	667 (15%)	4533

**Table 5.** Species richness (S), abundance (N), final slopes of the estimated species accumulation curves and completeness, based in two methods (see methods) for Araneae, Coleoptera and Hemiptera in Faial Botanic Garden (FAI) and Duque da Terceira Garden (TER). For the order Coleoptera, only the adult specimens were considered as there were no juveniles in our samples. Note: Chao1 was not possible to be calculated in cases without doubletons. (*) indicates higher level of biodiversity, based on the rarefaction method.

**Taxon**		**N**	**S (observed)**	**S (rarefied)**	**Slope**	**Completeness**	
						**Jackknife1**	**Chao1**
** Araneae **							
**Adults**	**FAI**	158	20	18.27	0.06	0.71	-
	**TER**	130	25	25*	0.08	0.66	-
**Total**	**FAI**	742	24	21.25	0.01	0.75	0.78
	**TER**	496	31	31*	0.02	0.70	0.79
** Coleoptera **							
**Adults**	**FAI**	178	21	21*	0.06	0.74	-
	**TER**	1465	95	20.4	0.01	0.95	-
** Hemiptera **							
**Adults**	**FAI**	151	14	14	0.07	0.89	-
	**TER**	397	32	22.60*	0.02	0.71	-
**Total**	**FAI**	1350	17	14.44	0.01	0.74	-
	**TER**	684	32	32*	0.01	0.80	-

**Table 6.** Rarity scores and Hill diversity metrics for the order Araneae, Coleoptera and Hemiptera in Faial Botanic Garden (FAI) and Duque da Terceira Garden (TER). For the order Coleoptera, only the adult specimens were considered as there were no juveniles in our samples. H´ -Shannon-Wiener index; D –Simpson index; d –Berger-Parker index. For the Berger-Parker index, (*) indicates higher level of biodiversity, based on randomisation test with 10,000 random partitions (Solow 1993).

**Taxon**		**Singletons**	**Doubletons**	**Rare sp** %	**Hill series**		
					**exp H**'	**1/D**	**1/d**
** Araneae **							
**Adults**	**FAI**	6.97	2.69	53	10.14	7.72	4.94*
	**TER**	9	3	48	11.36	5.72	2.60
**Total**	**FAI**	6.05	3.53	45	6.73	4.35	2.59*
	**TER**	9	5	45	6.31	2.77	1.70
** Coleoptera **							
**Adults**	**FAI**	7	2	43	11.04	7.69	3.71
	**TER**	9.78	3.37	65	9.32	5.94	5.57*
** Hemiptera **							
**Adults**	**FAI**	2	1	21.40	7.42	5.19	3.20*
	**TER**	6.91	2.79	43	11.22	7.19	2.90
**Total**	**FAI**	3.03	1.36	30.40	4.16	2.80	1.80
	**TER**	7	3	31.25	8.23	4.27	2.43*

**Table 7.** Number of endemic, native and introduced species (S) and individuals (N) of Araneae, Coleoptera and Hemiptera in Faial Botanic Garden (FAI) and Duque da Terceira Garden (TER).

**Taxon**		**Endemic**		**Native**		**Introduced**	
		**N**	**S**	**N**	**S**	**N**	**S**
** Araneae **							
**Adults**	**FAI**	32	1	25	2	101	21
	**TER**	4	1	60	5	54	24
**Total**	**FAI**	50	1	174	2	518	21
	**TER**	7	1	319	5	148	24
** Coleoptera **							
**Adults**	**FAI**	3	2	12	4	163	15
	**TER**	1	1	422	13	1034	78
** Hemiptera **							
**Adults**	**FAI**	45	3	92	10	14	4
	**TER**	27	1	127	14	243	17
**Total**	**FAI**	45	3	1279	10	26	4
	**TER**	27	1	132	14	525	17

**Table 8.** Dominant species of Araneae, Coleoptera and Hemiptera in Faial Botanic Garden (FAI) and Duque da Terceira Garden (TER). Colonisation status (E- endemic, N – native, I – introduced) and trophic status (P – predators; H – herbivores).

**Taxon**		**Dominant species**	**Colonisation status**	**Trophic status**	**Abundance**	**N_TOTAL_**
** Araneae **						
**Adults**	**FAI**	*Emblyna acoreensis*	E	P	32 (20%)	158
	**TER**	*Porrhoclubiona decora*	N	P	50 (38%)	130
**Total**	**FAI**	*Neoscona crucifera*	I	P	287 (39%)	742
	**TER**	*Porrhoclubiona decora*	N	P	292 (59%)	496
** Coleoptera **						
**Adults**	**FAI**	*Tachyporus nitidulus*	I	P	48 (27%)	178
	**TER**	*Sericoderus lateralis*	I	P	263 (18%)	1465
** Hemiptera **						
**Adults**	**FAI**	*Loricula elegantula*	N	P	47 (31%)	151
	**TER**	*Oxycarenus lavaterae*	I	H	136 (34%)	397
**Total**	**FAI**	*Cyphopterum adcendens*	N	H	725 (54%)	1350
	**TER**	*Cyphopterum adcendens*	N	H	281 (41%)	684

## Hosting institution

cE3c – Centre for Ecology, Evolution and Environmental Changes/Azorean Biodiversity Group and Universidade dos Açores - Departamento de Ciências e Engenharia do Ambiente, Rua Capitão João D ´Ávila, 9700-042 Angra do Heroísmo, Açores, Portugal
